# The Communication Between Intestinal Microbiota and Ulcerative Colitis: An Exploration of Pathogenesis, Animal Models, and Potential Therapeutic Strategies

**DOI:** 10.3389/fmed.2021.766126

**Published:** 2021-12-13

**Authors:** Yu Hu, Zhen Ye, Mingquan Wu, Yingqi She, Linzhen Li, Yujie Xu, Kaihua Qin, Zhipeng Hu, Maoyi Yang, Fating Lu, Qiaobo Ye

**Affiliations:** ^1^School of Basic Medical Sciences, Chengdu University of Traditional Chinese Medicine, Chengdu, China; ^2^Department of Pharmacy, Sichuan Provincial Orthopedic Hospital, Chengdu, China; ^3^Hospital of Chengdu University of Traditional Chinese Medicine, Chengdu, China; ^4^Guangzhou University of Chinese Medicine, Guangzhou, China; ^5^Health Preservation and Rehabilitation College, Chengdu University of Traditional Chinese Medicine, Chengdu, China

**Keywords:** ulcerative colitis, pathogenesis, intestinal microbiota, animal model, treatment strategy, fecal microbiota transplantation, probiotic, traditional Chinese medicine

## Abstract

Ulcerative Colitis (UC) is a chronic inflammatory bowel disease. The prolonged course of UC and the lack of effective treatment management make it difficult to cure, affecting the health and life safety of patients. Although UC has received more attention, the etiology and pathogenesis of UC are still unclear. Therefore, it is urgent to establish an updated and comprehensive understanding of UC and explore effective treatment strategies. Notably, sufficient evidence shows that the intestinal microbiota plays an important role in the pathogenesis of UC, and the treating method aimed at improving the balance of the intestinal microbiota exhibits a therapeutic potential for UC. This article reviews the relationship between the genetic, immunological and microbial risk factors with UC. At the same time, the UC animal models related to intestinal microbiota dysbiosis induced by chemical drugs were evaluated. Finally, the potential value of the therapeutic strategies for restoring intestinal microbial homeostasis and treating UC were also investigated. Comprehensively, this study may help to carry out preclinical research, treatment theory and methods, and health management strategy of UC, and provide some theoretical basis for TCM in the treatment of UC.

## Introduction

Ulcerative Colitis (UC) is a chronic inflammatory bowel disease (IBD) that involves the rectum and colonic mucosal layer, leading to superficial damage to the intestinal wall (1). Chronic diarrhea, fecal blood or rectal bleeding are the main clinical manifestations of these patients. Approximately 15% of UC patients develop severe illnesses (2). More importantly, chronic UC is associated with an increased risk of colorectal cancer. Yet the pathogenesis of UC has not been fully elucidated, it is mainly related to genetic, immunological, microbial and other risk factors. The intestinal microbiota is considered as a kind of “mysterious organization” in human body and has been proved to play an essential role in the pathogenesis of UC (3). In recent years, based on the development of high-throughput sequencing technology, related significant progress has been made in this research field, which is helpful for understanding the microbiota on human mucosal surface (4). Under physiological conditions, the interactions between bacteria, fungi, and other members maintain a dynamic balance called intestinal microbial homeostasis. Once the homeostasis is broken, the links between microbiota will change, resulting in a decreasing microbial diversity and increasing opportunistic pathogens (5). These changes further induce an abnormal immune response in the host intestine and eventually led to UC (6).

At present, anti-inflammatory and immunosuppressive therapies are the important treating method for UC. 5-aminosalicylic acid (5-ASA), corticosteroids, and thiopurines are commonly used drugs. However, steroid dependence and side effects of thiopurine make long-term use of the drug at high risk (7). Moreover, even with medication, 20–25% of patients eventually need surgery (8). As a consequence, it is imminent to find effective and safe treatment strategies. Of note, the therapeutic approaches aimed to improve microbial dysbiosis has shown great potential for the treatment of UC. Similarly, traditional Chinese medicine (TCM), as an important part of complementary and alternative medicines (CAMs), has been used in China for more than 2,000 years. At present, various scholars have begun to pay attention to the therapeutic effect of TCM on UC, and have made some progress in China (9, 10). Increasing evidence shows that the efficacy of TCM in the treatment of UC dependents on its effect on intestinal microbiota (11, 12).

**Figure 1:**
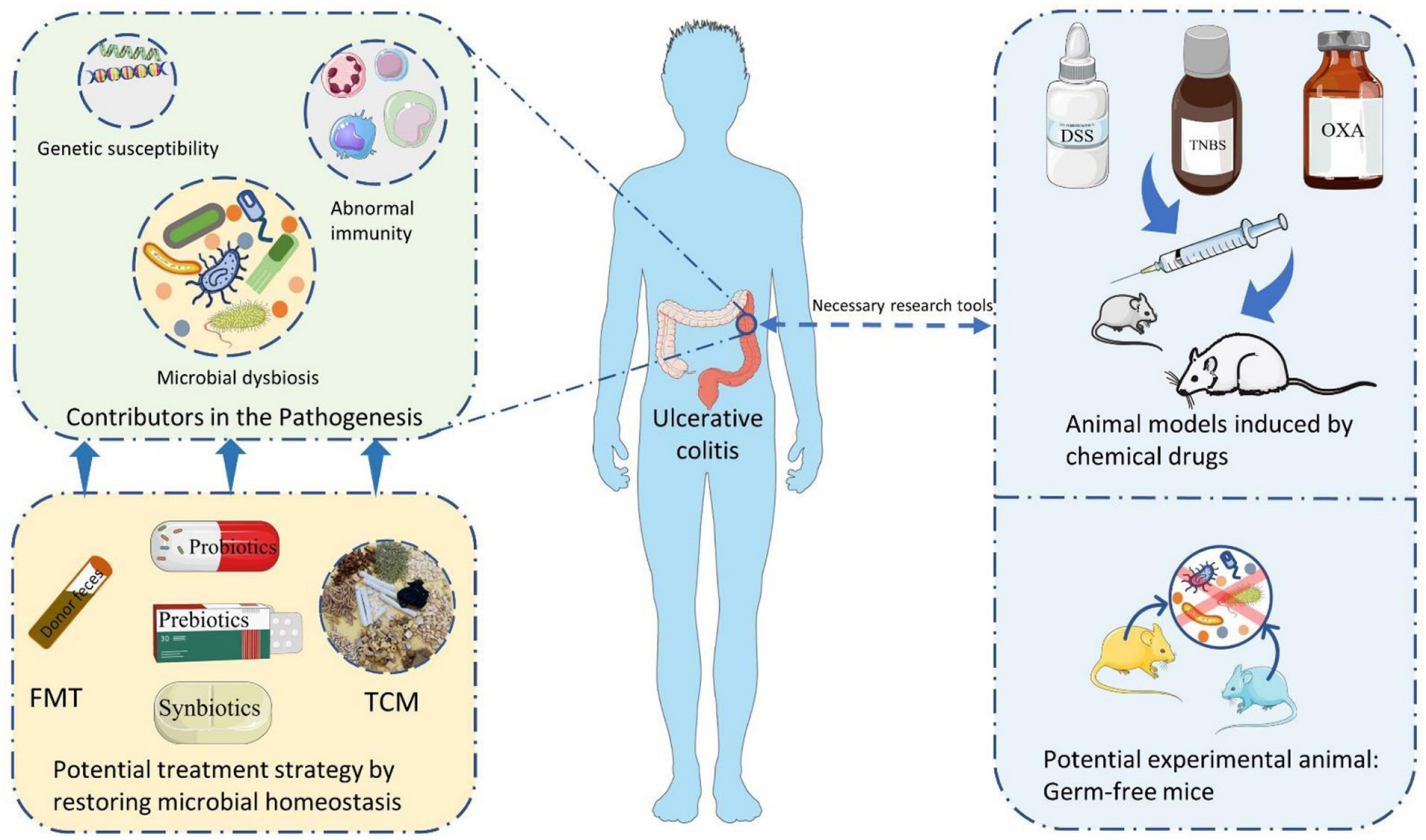
The summary of themes in this review. FMT, Fecal microbiota transplantation; TCM, traditional Chinese medicine; DSS, dextran sulfate sodium; TNBS, 2,4,6-trinitrobenzene sulfonic acid; OXA, oxazolone.

In this review, the essential components involved in the pathogenesis of UC are firstly summarized, and the critical role of intestinal microbiota is further discussed. Secondly, the potential of UC animal models applied in studying the intestinal microbiota is also addressed and evaluated. At last, potential therapeutic strategies which exerting a therapeutic role in UC by modulating the intestinal microbiota are exposed and revealed as much as possible (Figure 1). This work is expected to theoretical support and inspiration for the drug selection and development for treating UC.

## The Risk Factors With the Occurrence of UC

### Genetic Factors

Although certain studies have shown that the heritable risk of Crohn's disease (CD) is greater than that of UC, the risk of the disease in first-degree relatives of UC patients is still four times greater than that of the general population, suggesting that the risk of genetic factors remains a contributor to the occurrence of UC (13, 14). The first genome-wide association studies based on IBD identified IL23R, a gene encoding the pro-inflammatory cytokine interleukin (IL)-23, whose abnormalities and mutations are associated with the development of CD and UC (15). To date, more than 240 risk loci have been identified to be associated with IBD (16). It was interesting that only 4–7% of UC occurrence can be explained through known risk loci, but genetic factors appear to be more important for early IBD that develops in children (17, 18).

The disease-associated loci of involving genes with different functions, such as the innate and adaptive immune systems, cytokine signaling, lymphocyte activation, and response function to microbial molecules (17). For example, variants in CARD9, a bridging protein involved in antifungal innate immunity, enhance the host response to the fungus and increase the production of inflammatory cytokines, and is also considered as one of the genetic risk factors for CD and UC (19). Moreover, the ADCY7 gene is expressed in hematopoietic cells and encodes a protein, adenylate cyclase 7, which converts ATP to cAMP to participate in the regulation of host innate and adaptive immunity (20, 21). And it is reported that the missense variant of ADCY7 increases the risk of UC. For other IBD risk loci, a complete description was given in the research article published in 2017 (16).

So far, the genetic risk of CD has been well studied, and a large number of studies have shown a strong genetic predisposition for CD. For example, disorders of the NOD2 gene have been identified as an essential causative factor for CD occurrence (22). At the same time, researches on the genetic risk of UC and IBD is continuing, which not only facilitates the development of the pathogenesis of IBD and the differentiation of IBD subtypes but also contributes to the updating of therapeutic targets and drugs or programs for the disease.

### Immunological Factors

Genetic, environmental and microbial factors have been identified as risk factors of UC (22). However, UC is ultimately linked with immune abnormalities, which indicated immunological factors may be the central link (1). The human immune system can be divided into innate immunity and adaptive immunity according to functions, and the available evidence points to the involvement of both innate and adaptive immunity in the pathogenesis of UC (23).

The innate immune response is the first line of defense against any attack, and the neutrophils, dendritic cells or macrophages that mediate innate immunity has been confirmed to be involved in the pathogenesis of UC. The earliest feature of intestinal inflammation is the infiltration of neutrophils into the mucosa and epithelium, and is present throughout the period of active inflammation of the intestine. The tissue and epithelial barrier disruption are caused by neutrophils with oxidative and proteolytic damage, as well as promote the release of pro-inflammatory cytokines to perpetuate inflammation (24). Dendritic cells are also involved in the expression of Toll-like receptors (TLRs) and the recognition of microbiota (25). Increased activation and sensitivity of mature dendritic cells and significantly higher the level of TLR2 and TLR4 expressed by mucosal dendritic cells are observed in UC patients, which lead to abnormal activation of signaling pathways such as the nuclear factor κB (NF-κB) to promote the inflammatory cascade (26). Macrophages can be polarized into classically activated pro-inflammatory or alternatively activated anti-inflammatory macrophages depending on the stimulus (27). The pro-inflammatory macrophages are induced by pathogen-associated molecular patterns and involved in the production of pro-inflammatory cytokines such as tumor necrosis factor-α (TNF-α), IL-1β and IL-6 to intensify inflammation (28). In addition, the polarization of pro-inflammatory macrophages has been found to promote the development of colitis. In conclusion, excessive abnormalities in innate immunity and the occurrence of inflammatory cascade aggravate the emergence and persistence of local inflammation in the colon, which is closely associated with the occurrence of UC.

After recognizing of antigens, the dendritic cells and macrophages will present antigens to T cells and B cells, leading to the activation of adaptive immunity (29). And the abnormalities of adaptive immunity are another risk factor of UC (30). UC is thought to be a disease mediated by T helper type (Th) 2 cells, which are involved in the secretion of IL-4, IL-5 and IL-13. Published studies have shown the increase in IL-5 and IL-13 secretion in UC patients, with IL-13 affecting the intestinal epithelium and disrupting tight junctions to cause an inflammatory state (31, 32). In addition, abnormalities in Th9 and Th17 cells provide evidence for the involvement of adaptive immunity in UC. Th9 cells are associated with the release of IL-9, which can inhibit the repair of intestinal epithelial cells and increase the concentration of TNF-α (33). Similarly, Th17 cells are also involved in the release of pro-inflammatory cytokines such as IL-17A, IL-17F, IL-21 and IL-22, and increased Th17 cells expression has been observed in UC patients (34, 35). In summary, current evidence suggests that both innate immunity and adaptive immunity are critical in the pathogenesis of UC (1).

### Microbial Factors

#### The Role of Intestinal Bacteria in the Pathogenesis of UC

The intestinal microbiota mainly settles in the gastrointestinal tract of humans, and bacteria occupy the primary advantage in the composition (36). The intestinal bacteria are comprised of three types: anaerobic bacteria, facultative anaerobic bacteria and aerobic bacteria, with anaerobic bacteria being the dominance (37). At the phylum level, the intestinal bacteria are mainly composed of *Firmicutes, Bacteroides, Proteobacteria* and *Actinobacteria* (38). Under physiological conditions, intestinal bacteria play an essential role in stimulating the absorption of nutrients and minerals, breaking down protein compounds, synthesizing amino acids and vitamins, promoting intestinal cell renewal, and maintaining immune function (39).

Heredity, age, environment and dietary structure can influence the composition of intestinal bacteria. For example, a high-fat diet reduces the abundance of *Bifidobacterium spp* in the intestinal tract of mice (40). Various types of studies have shown that the abundance of *Bacteroides* in the elderly is greater than that of the young (41). Other studies reported that the intestinal bacteria's amount and diversity of UC patients are significantly decreased (42). At the same time, the changes in flora composition were also found in the pathological state. At the phylum level, the abundance of *Bacteroidetes* and *Proteobacteria* increased, while the abundance of *Firmicutes* decreased (5). In specific microbes, the number of beneficial bacteria such as the *Roseburia spp* and *lactobacillus* in the intestine decreased, while the number of destructive bacteria such as *Escherichia coli, Bacteroides fragilis*, and *Helicobacter* increased (5, 43, 44). So far, many experts believe that the dysbiosis of intestinal microbiota can lead to the dysregulation of the immune response to bacterial antigens, and ultimately leads to the occurrence of IBD (45, 46).

The intestinal epithelial barrier is mainly composed of the intestinal epithelial cells, tight junctions (connecting the epithelial cells), goblet cells, and mucus (secreted by goblet cells). This barrier is the first defensing phase to ensure the normal physiological function of the intestinal tract and prevent pathogenic microbes from crossing the intestinal mucosa. As an integral part of the barrier, intestinal mucus can limit the direct contact between host and intestinal bacteria, promote bacteria clearance, and inhibit inflammation and infection (47). Furthermore, dysbiosis of intestinal microbiota can affect the function of epithelial barrier. Firstly, the tight junction is damaged, resulting in increased intestinal mucosa permeability (48). Secondly, the dysbiosis of bacteria will significantly impact intestinal mucus. For example, *Akkermansia muciniphila* and *Enterorhabdus mucosicola* can degrade intestinal mucus and proliferate in the mucus layer (42), *Escherichia coli* and *Gardnerella* can form adherent biofilms on the surface of intestinal epithelium, destroy intestinal mucus, and allow other commensal bacteria to migrate to the mucosa (49). Muc2 protein, secreted by goblet cells, is the primary source of mucus. This protein is mediated by intestinal microbes to maintain the balance of the mucus layer. However, loss of goblet cells and Muc2 protein is a typical feature of epithelial barrier changes after the dysbiosis of intestinal bacteria in UC patients (50).

**Figure 2:**
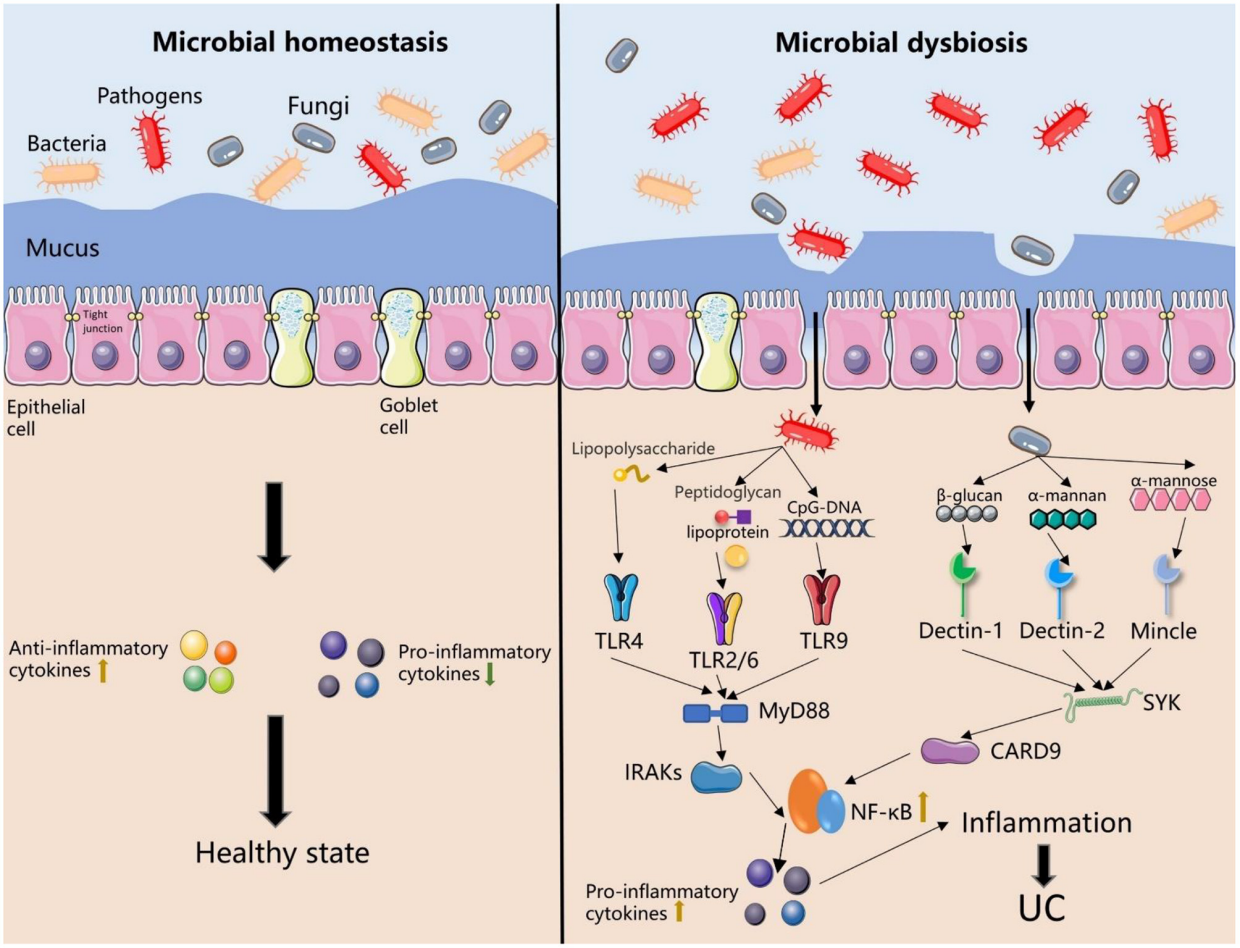
The dysbiosis of intestinal microbiota (bacteria and fungi) results in the occurrence of UC. Under the influence of risk factors, intestinal microbial homeostasis is disrupted. The dysbiosis of microbiota leads to changes in the tight junctions between intestinal epithelial cells, damage of intestinal epithelial barrier, disruption of the mucus layer, and an increase of intestinal permeability. Symbiotic microorganisms and pathogens cause direct damage to the intestinal wall. Furthermore, the main components of the bacterial cell wall (lipopolysaccharide, peptidoglycan, and lipoprotein) are recognized by TLRs, and C-type lectin receptors recognize the components of the fungal cell wall (β-glucan, α-mannan, and α-mannose). After recognition of the antigen, these receptors induce downstream signaling and activate the NF-κB signaling pathways, further induce abnormal immune responses in the host and ultimately leading to the occurrence of UC.TLR, Toll-like receptor; MyD88, Myeloid differentiation factor 88; IRAK, interleukin-1 receptor-associated kinase; NF-κB, nuclear factor κB; Mincle, Macrophage-inducible C-type lectin; SYK, spleen tyrosine kinase; CARD9, caspase recruitment domain family member nine.

When the epithelial barrier is disrupted, symbiotic bacteria are allowed to flow in the epithelial layer. Invasion of pathogenic bacteria and opportunistic pathogens activate the host's maladaptive immune response (51). TLR is a protein molecule involved in nonspecific immunity, while lipopolysaccharide, a component of the outer membrane of Gram-negative bacteria, can bind to TLR4 (52). Similarly, peptidoglycan, lipoprotein, and lipoteichoic acid in the bacterial cell wall can also bind to TLR2/TLR6 complex. TLR9 responds to non-methylated bacterial DNA (CpG-DNA) (53). After recognizing the components of the bacterial cell wall, TLRs interact with Myeloid differentiation factor 88 (MyD88), up-regulated the activation signal of IL-1 receptor-related kinase (IRAK) of related member, activated the NF-κB signaling pathway, released pro-inflammatory cytokines, resulting in abnormal intestinal inflammation and occurrence of UC (Figure 2) (54).

As well, dysbiosis of intestinal microbiota also affects the regulation of bacterial metabolites (55). Short-chain fatty acids (SCFAs) are produced by symbiotic bacteria that digest dietary fiber in the intestine, including acetate, propionate, and butyrate, etc. (56). SCFAs can maintain the balance of regulatory T cells number to regulate intestinal inflammation (57). It is worth noting that the disorder of SCFAs' generation occurs in UC patients, which is related to the significant reduction of butyrate-producing bacteria (5). Therefore, the dysbiosis of intestinal bacteria further leads to the disorder of its metabolites and products, destroys normal immune response of the host intestine, and is also an important factor leading to UC.

#### The Role of Intestinal Fungi in the Pathogenesis of UC

Fungi are also normal inhabitants of the human intestine. In healthy individuals, the number of intestinal fungi only accounts for 0.1% of intestinal microorganisms, among which the most dominant fungi are *Candida, Saccharomyces* and *Cladosporium* (4). The interaction between symbiotic fungi in the intestine and the other microbial members remain balanced, but risk factors can cause changes in the composition of fungi in the human intestine. High-carbohydrate diets increase the number of *Candida albicans* in the intestine, while high-protein diets are the opposite. And meat-based diets promote the abundance of *Penicillium* fungi (58, 59). An analysis showed different intestinal fungal spectrums in UC patients and healthy people, and the diversity of intestinal fungi in UC patients was significantly reduced (60). In terms of its composition, intestinal fungi of UC patients also changed. The abundance of *Candida albicans* increased, and the abundance of *Wickerhamomyces* fungi was positively correlated with the severity of UC patients (61). *Saccharomyces cerevisiae*, a fungus that can stimulate the release of IL-10 to inhibit intestinal inflammation, and its abundance is absolutely decreased in the acute phase of intestinal inflammation (62).

Intestinal fungi imbalance can reduce host immune tolerance and activate abnormal immune responses. The main component of the fungal cell wall, β-glucan, α-mannan and α-mannose, can be identified by TLRs and C-type lectin receptors (Dectin-1, Dectin-2, and Mincle) (63). Dectin-1 is a key molecule involved in fungi immune response, and the polymorphism of the Dectin-1 gene is closely related to the severity of UC. Mice without Dectin-1 were more susceptible to dextran sulfate sodium (DSS)-induced colitis (63). After recognition, spleen tyrosine kinase (SYK) and caspase recruitment domain family member 9 (CARD9) were activated, thereby activating the NF-kB pathway, releasing pro-inflammatory cytokines, and inducing inflammatory response (Figure 2) (64).

In addition, the DSS-induced mouse antifungal drug experiment reported that antifungal drugs could aggravate the inflammation response in mice (65). The expression of tumor necrosis TNF-α and IL-17A in the colon of UC patients is positively correlated with *Wickerhamomyces* and *Penicillium* fungi (61). These studies also provide evidence that intestinal fungal dysbiosis is associated with the occurrence of UC.

#### Intestinal Microbial Homeostasis and UC

Intestinal microbiota is a complex and extensive system composed of bacteria, fungi, archaea, viruses and protozoa, *etc*. Interactions between the microbiome maintain homeostasis, help host resist pathogen infection, and promote host immunity and health (66).

The interaction between intestinal fungi and bacteria plays a key role in maintaining intestinal microbial homeostasis. A study found that after antibiotic treatment, the number of intestinal fungi in mice increased as bacteria continued to decrease. This change returned to the initial state after the antibiotics stopped (67).

However, after the use of antifungal drugs, the decrease of intestinal symbiotic fungi promotes the growth of pathogenic bacteria in the intestinal tract, thereby aggravating intestinal inflammation (68). Bacteria can limit the effective colonization of fungi in the intestinal tract by producing antifungal compounds and competing nutrients. This phenomenon reflects the competitive relationship between bacteria and fungi due to the limited intestinal resource. In fact, there is also a synergistic relationship between them (69). *Candida albicans* is the most common human infectious fungi, mainly affecting immunologically impaired individuals (70). In another study, researchers found that the harmful effects of *Candida albicans* depended on the presence of colistin-sensitive bacteria in the intestine (71). At the same time, *Candida albicans* can form mixed biofilms with other intestinal bacteria, which can surround anaerobic bacteria to protect them from the effects of an oxygenated environment (72).

Under different interactions, intestinal microbiota maintains balance and stability in the intestine. Affected by risk factors, the dysbiosis of intestinal bacteria and fungi leads to susceptibility of the host to UC (73). The positive and negative correlations between intestinal bacteria and fungi in UC patients are higher than those in healthy individuals (62). Therefore, the disruption of intestinal microbial homeostasis is tightly related to the occurrence of UC. With the increase of relevant evidence, the relationship between microbial homeostasis and the pathogenesis of UC is increasingly obvious.

### The Importance of the Intestinal Microbiota in UC's Occurrence

Abnormalities of the immune system are certainly important for the pathogenesis of UC, which is also a major reason for the appearance and persistence of local inflammation in UC patients. However, except for the immune system, the mucus and epithelial barriers are the first physical and chemical lines of defense protecting the intestinal epithelium from pathogens and antigens. Mucus barrier disorders in the colon are also a possible cause of UC. Moreover, a recent study has shown that the disorders of mucus barrier were not related to local inflammation and immune response, but the response of goblet cells to microbial alterations (74). In addition, defects in the mucus barrier occur early in the onset of UC, further leads to increased intestinal permeability and exposure to antigens, thus activating abnormal immune responses (22, 23). This suggests that it was necessary to consider other risk factors. Notably, there is growing evidence that dysbiosis of intestinal microbiota is associated with disruption of the intestinal barrier, increased permeability, and increased antigen exposure.

Bacteria occupy the absolute advantage of intestinal microbiota, but fungi, viruses, and archaea are also important parts of the intestinal microbiota. Microbiota maintains a dynamic balance through complex interactions and connections, and the homeostasis is a key to maintaining host healthy. The composition of intestinal microbiota and the interactions between microorganisms are often altered by the risk factors such as genes, eating habits, unhealthy lifestyles, and drug abuse. For example, a long-term high-fat diet thins intestinal mucus, increases intestinal permeability, and impairs the tight junctions of intestinal epithelial cells, leading in intestinal barrier dysfunction (75). Additionally, the intestinal barrier function tends to deteriorate as the body matures (76). Increased intestinal permeability and breakdown of the intestinal barrier allow bacteria to readily pass past the epithelium and colonize the mucosa, hence amplifying their effect on the intestine (77). Although intestinal commensal microbiota contributes to the regulation of intestinal epithelial and immune function in the physiological state, risk factors such as diet, environment, and age can also result in dysbiosis of the intestinal microbiota, as revealed by decreased diversity and changes in specific microbial species and abundance (3). Inequality within each microbial community and changed interactions contribute to overall microbial homeostasis imbalance (78). Reduced probiotic bacteria and a rise in pathogenic and opportunistic pathogenic bacteria are significant aspects of microbial homeostasis imbalance in UC patients (79–81). The intestinal microbiota can directly interact with the immune system of the body (82). Certain *Clostridium* bacteria demonstrate pro-inflammatory properties by penetrating and mucosalizing the intestinal mucosa. Similarly, increasing *bifidobacteria* abundance has been shown to stimulate Th1 cell-mediated immune responses (83). Additionally, TLRs identification of bacterial and fungal surface components results in excessive activation of TLRs signaling pathways, triggering innate and adaptive immunity and chronic inflammation when the gut barrier is disrupted and pathogenic bacteria colonize. The metabolites generated by intestinal microbiota play a role in modulating host immune responses, such as SCFAs and bile acids, which are critical for sustaining anti-inflammatory effects and safeguarding intestinal barrier function (84, 85). Likewise, a decrease in the absolute abundance of SCFAs-producing bacteria is another unique symptom of microbial dysregulation in UC patients. Thus, the combination of reduced intestinal barrier function, microbial homeostasis imbalance, and lack of beneficial microbial products results in disruption of the intestinal epithelium and an abnormal immune response, which is a significant contributor to the development of UC.

The intestinal microbiota is a very complex and extensive system. Although the mechanisms of bacteria and fungi in the occurrence of UC are increasingly obvious, members such as viruses and archaea are also related to UC. Therefore, more in-depth studies are needed to explore the relationship between the intestinal microbiota and UC. At the same time, drugs and treatment methods focusing on restoring intestinal microbiota homeostasis may be the key to the treatment of UC.

## Common Animal Models of UC

Establishing an appropriate animal model helps to study the pathogenesis of UC and investigate potential therapeutic drugs. UC animal models can be established with chemical drug induction, pathogen colonization, genetic engineering modification, and adoptive cell transfer (86). Notably, due to the limitations of modeling methods and prices, experimental colitis induced by the chemical drugs has become the most common animal model of UC (87). DSS, 2,4,6-trinitrobenzene sulfonic acid (TNBS) and oxazolone (OXA) are three commonly used chemical drugs. Therefore, it is necessary to review the advantages and limitations of these three models to provide a scientific basis for the reasonable selection of UC animal models.

### DSS

DSS is normally used to induce experimental colitis by dissolving it in water and provided ad libitum for several days to animals. It is generally believed that DSS can induce acute UC in SD rats and Winstar rats at a concentration of 2–5% added to drinking water for 5–9 days. At the same time, adding 3–5% of DSS in drinking water for 5–8 days can induce acute UC in C57BL/6 mice and BALB/C mice (88). Multiple repeated drinking cycles are required if model is necessary to induce chronic UC (89). DSS can cause complete loss of intestinal epithelial cells to destroy the epithelial barrier. The lamina propria and submucosa are subsequently exposed to antigens and microbiota in the intestinal lumen, which eventually induced inflammation (90). During the administration of DSS, animals showed weight loss, thickening of stool, bloody stool and diarrhea *etc*. (91). Histopathological changes such as epithelial denuding, loss of mucin and goblet cells, submucosal edema, hyperemia and erosions, infiltration of inflammatory cells in the lamina propria and loss of crypt structures could be observed (92, 93).

The DSS-induced UC model has the characteristics of simple administration, easy control of drug dose and duration. Moreover, researchers can design acute or chronic experimental colitis by controlling the dose and duration of administration. Meanwhile, due to the induction of severe intestinal ulcer in animals, this model is also been interpreted as the animal model closest to human UC (94). Similarly, DSS-induced model also showed changes in the intestinal microbiota. The decrease in microbial diversity and abundance of beneficial symbiotic bacteria (*Lactobacillus* and *Alistipes*), and the increase of the abundance of pathogenic bacteria (*Oscillibacter, Streptococcus*, and *Escherichia–Shigella*) are similar to the changes of intestinal microbiota in UC patients (87, 95), which makes it also has advantages in studying the effects of drugs on the intestine microbiota. However, DSS-induced UC model also has obvious limitations. It is reported that DSS-induced inflammatory response began to ease naturally after 7 days, and the changes in the intestinal microbiota returned to normal levels after 21 days (87, 95). Besides, the sensitivity of certain types of mice to DSS is also different. After 5 days of acute administration, C57BL/6 mice induced colon inflammation, while BALB/C mice recovered simultaneously (94). Since UC itself is a chronic disease, DSS-induced model still lacks sufficient representation for UC.

### TNBS

Rats were first used in TNBS-induced experimental colitis modeling. 5-30 mg TNBS into 0.25 ml of 50% ethanol, and the colon ulcer and inflammation in rats were successfully induced by intraluminal drip administration. The BALB/C and C57BL/6 mice are also used for colitis models by 0.3–5.0 mg of TNBS mixed with 50% ethanol (96). In TNBS-induced animal model, the use of ethanol is not only a solvent or carrier but also can destroy epithelial barrier of animals. TNBS is a hapten reagent that induces the immune response of T cells to tentacle proteins and luminal antigens through acute oxidative stress, resulting in intestinal wall necrosis (97). After administration of TNBS, animals showed bloody diarrhea and weight loss. Histopathological changes can be observed in intestinal wall thickening, edema, bleeding, and ulcer (98). Chronic TNBS colitis is manifested with diffuse necrosis of the intestinal wall, involving the mucosa, submucosa and muscle layer. At the same time, there are significant edema and immune cell infiltration in submucosa (99).

The TNBS-induced colitis model has the advantages of rapid disease development, localized colon injury and low cost. Compared with DSS-induced UC model, animal inflammation does not begin to relieve until the 15th day, which seems to be more conducive to researchers to ensure the accuracy of their research results (97). However, TNBS-induced animal model is associated with the immune response mediated by Th1 cells, which is considered closer to the immunological changes of CD. Moreover, the intestinal microbial changes in TNBS-induced mice showed a decrease in α-diversity, possibly beneficial bacteria decreased and harmful bacteria increased, similar to those in patients with Crohn's disease (100). Interestingly, some experts believe that it causes intestinal ulcer in animals to be similar to UC (101). Hence, it is still doubtful whether the TNBS-induced colitis model can represent human UC.

### OXA

OXA is also a hapten reagent that needs to be administered with ethanol to induce colitis in animals (102). Before administration, 3% OXA (100–150 μl) was dissolved in 40–50% ethanol to sensitize the skin of mice and induce experimental colitis (103). The sensitivity of different strains of mice to OXA is also different. SJL/J mice and C57BL/10 mice are highly susceptible to OXA, but C57BL/10 mice are more resistant than SJL/J mice. BALB/C mice are more suitable for inducing chronic colitis (104).

The OXA-induced mice have symptoms of diarrhea, bloody stool, and weight loss. Histopathological changes are mainly characterized by epithelial cell loss, inflammatory cell infiltration, edema, and occasionally crypt abscess (105). As a kind of hapten reagent, OXA-induced colitis in mice is closely related to Th2 cell-related immune response, which is similar to human UC (91, 106). Compared with other animal models induced by chemical drugs, The OXA-induced model is more suitable for studying the pathology of UC. However, due to the high mortality rate of OXA, it is rarely applied in practical research (94, 105). In experimental animals, whether OXA can cause microbial disorders similar to human UC still needs more research.

### The Role of Microbiota in Different UC Animal Models

As is well known, the application of drugs is the key to successful modeling of chemically induced animal models. However, intestinal microbiota has also been found to play a role in different animal models. A study found that during the induction of colonic inflammation in mice with DSS, an increase in the proportion of cells expressing CD11c and TLR4 in the mesenteric lymph nodes of mice was consistent with an increase in the number of *Enterobacteriaceae* and *Akkermansia* on the colonic mucosa, implying that the response of the immune system to microbial recognition and the induction of inflammatory responses (107). Similarly, in TNBS and OXA-induced inflammation models, the abundance of Sulfate-reducing bacteria, which produce hydrogen sulfide that damages the colon, causes epithelial damage and inflammation, and induces a Th17 cell-associated immune response, was increased (108, 109).

All of these studies provide that microbiota is also vital in UC animal models. However, there are existed those different conclusions were from other experts. Gancarcikova et al. found the absence of microbiota did not affect the inflammatory effects of DSS when exposed antibiotic-treated germ-free mice to DSS (110). Another study found that in the absence of intestinal microbiota, the function of the intestinal barrier was also impaired although intestinal inflammation was significantly reduced in animals exposed to DSS (111). Obviously, the intestinal microbiota is a very complex organization, and different microorganisms play different roles. Therefore, further studies on the specific effects of microbiota in animal models are still necessary to resolve the current controversies, but this does not affect the use of animal models as a tool for UC research in subsequent studies.

### Selection of Animal Models

The establishment of animal models is necessary to study the pathogenesis and pathological changes of UC. So far, the DSS-induced mice model has been most widely used. The intestinal microbiota of mice showed similar diversity to humans at the levels of *Firmicutes, Bacteroidetes*, and *Proteobacteria*. And the DSS-induced model is considered to be similar to the changes in the intestinal microbiota in UC patients. Therefore, DSS may be the current advantageous animal model for studying the relationship between intestinal microbiota and UC. However, the DSS-induced model may still be a limited result of obvious inflammation and rapid recovery of intestinal microbiota disorder, which still needs to be improved and developed.

Notably, many researchers have begun to use germ-free mice as an important tool to mimic the close relationship between intestinal microbiota and host (112). Studies ranged from single microorganisms to whole microbiota can be carried out in germ-free mice (23). For example, the intestinal bacteria highly coated with immunoglobulin A in IBD patients were isolated and cultured, and then transplanted into germ-free mice, which were eventually found to exhibit a high susceptibility to DSS-induced colitis (113). Consequently, germ-free mice may be a promising UC animal model, and the studies based on germ-free mice are also more conducive to revealing the specific link between intestinal microbiota and UC pathogenesis.

## The Medication of UC

The disease progression with outpatient medications can be managed successfully in most UC patients (114). The choice of specific medications should be evaluated according to the severity of disease, with 5-ASA preferred for patients with mild to moderate UC, and corticosteroids for patients who do not respond to or are intolerant of 5-ASA medications (1, 115). For patients with moderate to severe UC, systemic corticosteroids are the first-line induction therapy. Thiopurines are indicated for patients with steroid-refractory or steroid-dependent UC (116, 117). Moreover, current guidelines also recommend thiopurines as corticosteroid-free maintenance therapy (118). The mechanism of action, advantages and limitations of each of the three classes of drugs are different and compared (Table 1).

**Table 1:** The difference of three types of commonly used drugs in treating UC.

	**5-ASA**	**Corticosteroids**	**Thiopurines**
Applicable stage	Mild to moderate UC patients	Mild to moderate UC patients who are unresponsive or intolerant to 5-ASA treatment Moderate to severe UC patients	Steroid-dependent and Steroid-refractory UC patients
Mechanism of action	Acting on the colonic epithelium, exerting local mucosal anti-inflammatory effects	Inhibition of gene expression in the nucleus to suppress pro-inflammatory signaling pathway activation and limit immune cell translocation to sites of inflammation	As an immunosuppressant that inhibits inflammatory gene expression.
Advantages	Safety and Efficacy are proven	Available for UC patients who do not respond to 5-ASA therapy	Available as a maintenance treatment option for UC patients
Limitations	Some patients do not respond to 5-ASA treatment Not suitable for the treatment of moderate to severe UC patients	Long-term use will increase the risk of steroid dependence and steroid refractory	Obvious adverse events and serious potential side effects

*5-ASA, 5-aminosalicylic acid; UC, ulcerative colitis*.

### 5-ASA

The use of 5-ASA dates back to 1941 when sulfasalazine (SASP) was used for the treatment of UC. SASP is composed of 5-ASA linked to sulfapyridine via a diazo bond, which can be cleaved by bacteria in the colon. After colonic bacterial azoreductase enzyme cleaves the diazo bond, 5-ASA achieves a high intraluminal concentration in the colon, makes it the active moiety of SASP (119, 120). To date, mesalazine and SASP are the main 5-ASA drugs used in the treatment of UC.

5-ASA acts on the colonic epithelium and exerts local mucosal anti-inflammatory effects by inhibiting cyclooxygenase and lipoxygenase, which subsequently leads to a decrease in prostaglandin and leukotriene production (121, 122). It has also been reported that 5-ASA inhibits the activation of the NF-κB signaling pathway, which promotes the transcription of pro-inflammatory cytokines and is an important mechanism in the pathogenesis of UC (123). In addition, 5-ASA also inhibits the function of active lymphocytes, macrophages, and natural killer cells in the inflammatory process, which can scavenge the reactive oxygen metabolites (122). In conclusion, the local mucosal anti-inflammatory effect of 5-ASA is the main mechanism of action for treating UC.

Both American Gastroenterology Association and European Crohn's and Colitis Organization recommend 5-ASA as the first-line therapy for mild to moderate UC (116, 117). And the 5-ASA compound is a pillar for patients with mild to moderate ulcerative colitis, both as induction and maintenance therapy. Studies have shown that one-third of mild to moderate UC patients achieved clinical remission and half of the patients had mucosal healing after 8 weeks of oral 5-ASA treatment (124). Moreover, up to three-fifths of patients showed significant clinical remission and endoscopic improvement with 5-ASA maintenance therapy, which strongly supports the effectiveness of 5-ASA (125). In terms of specific drug use, there was no significant difference in efficacy between mesalazine and SASP, but patients with SASP often experienced adverse events (124, 125). However, 5-ASA still has a high safety profile compared to corticosteroids and thiopurines, and long-term 5-ASA therapy may have a preventive effect on colorectal cancer (126, 127). Notably, several studies have demonstrated that 5-ASA (including SASP and mesalazine) can restore the microbial diversity and the abundance of beneficial bacteria and fungi, reduce the abundance of pathogenic bacteria, and increase the production of SCFAs in experimental colitis and UC patients, which implying that 5-ASA also has the potential to modulate gut microbial homeostasis (80, 128, 129). Unfortunately, 5-ASA is not available for all UC patients. First, there are still some patients who do not respond to or are intolerant of 5-ASA therapy, and such patients usually dependent on corticosteroids. Second, for patients with moderate to severe UC, guidelines suggest that 5-ASA should not be used for induction or maintenance therapy (116, 117). Thus, corticosteroids, thiopurines, and even advanced therapies are in development after 5-ASA.

### Corticosteroids

Corticosteroids are used in patients with mild to moderate UC who are unresponsive or intolerant to 5-ASA therapy. In addition, corticosteroids are the treatment of choice for patients with moderate to severe UC. The first use of corticosteroids in treating UC was reported in 1955, with the surgical resection rate of the colon was significantly lower in UC patients treated with cortisone than in the placebo group (130). After that, the first generation of corticosteroids such as prednisone and hydrocortisone began to be widely used in UC. Corticosteroids act in the cell nucleus and ultimately play a role in regulating the immune response by inhibiting gene expression during transcription, down-regulating the production of transcription factor NF-κB and the expression of pro-inflammatory cytokines, and causing a decrease in the expression of adhesion molecules to limit the transfer of immune-inflammatory cells to inflammatory areas (131–133).

Since the immunosuppressive effects of first-generation corticosteroids are non-specific, inevitably, other body parts besides the intestinal inflammatory sites are also affected. As a result, up to 90% of UC patients experienced adverse effects after corticosteroid treatment (134), which led to the creation and development of second-generation corticosteroids such as beclomethasone dipropionate and budesonide. Compared to first-generation corticosteroids, second-generation corticosteroids can target the site of inflammation to exert local anti-inflammatory effects, to potentially reduce systemic corticosteroid concentrations (135). Similarly, the use of second-generation corticosteroids has an overall better safety profile and a reduced incidence of adverse events (136). However, all corticosteroids are absorbed by the body to some extent, and this leads to a continued occurrence of adverse events. In a study evaluating the safety of budesonide MMX, a novel Multimatrix formulation of budesonide, adverse events were reported in 31.8% of patients (137). In conclusion, as hormonal drugs, the therapeutic risk of corticosteroids rises with increasing dose and duration of exposure (138). In addition, steroid dependence is an issue that has to be considered (139). Therefore, corticosteroids are not recommended as the first choice for maintenance therapy, and corticosteroid use should be tapered or discontinued after clinical remission.

### Thiopurines

The first application of thiopurine for UC was in 1962 (140). To date, thiopurines are the most commonly used drug for maintenance treatment of UC after 5-ASA (141). Thiopurines are recommended for maintenance therapy without corticosteroids. In addition, the use of thiopurines is necessary for patients with moderate to severe UC who have developed steroid-refractory or steroid-dependent. Thiopurine analogs include azathioprine, mercaptopurine, and thioguanine, of which thioguanine is considered an atypical thiopurine drug, and is used only in those that have failed to respond to mercaptopurine and azathioprine in a few countries and regions (121). As a type of immunosuppressants, the mechanism of action of thiopurines may be the incorporation of their pharmacologically active metabolite, 6-thioguanine nucleotides, into DNA or RNA as false purine analogs, which lead to DNA damage, cell cycle arrest and apoptosis, and inhibition of nucleotide and protein synthesis (142). Ultimately, the expression of inflammatory genes can be inhibited (142).

Although thiopurines are thought to play a corticosteroid-sparing role in the treatment of steroid-dependent UC patients, it remains controversial in using thiopurines. Firstly, thiopurines have significant toxic effects, including bone marrow suppression, impaired red blood cell regeneration, and death in rare cases (143). Secondly, the use of thiopurines may increase the incidence of lymphoma and non-melanoma skin cancer in patients (144). Therefore, the treatment of thiopurines needs to be effective in a way that ensures a reduced risk of side effects, which often requires a rigorous evaluation by the physician.

### Potential Treatment Strategies of UC

Undoubtedly, 5-ASA, corticosteroids, and thiopurines have controlled the course of UC, and even saved the lives of a large number of UC patients. But the limitations of these drugs are also obvious and led to the search for more effective and safer drugs, which is an important reason for the rapid development of more advanced treatment strategies such as biologics and small molecules products. In addition, the microbial dysbiosis often occurs at the early stage in the occurrence of UC, suggesting that the therapeutic strategy based on restoring the microbial balance may be beneficial (145). Therefore, fecal microbiota transplantation (FMT), probiotics, prebiotics and synbiotics have become potential therapeutic approaches for UC and researched in fronter. It is also mentioned that TCM has potential advantages in the treatment of UC and should be paid more attention to (146).

#### FMT

FMT, originally used in the treatment of *Clostridium difficile* infection, is a method of regulating microbial dysbiosis by transplanting healthy donors' feces into patients (147). In 2013, the U.S. Food and Drug Administration officially approved FMT as a clinical treatment for recurrent or refractory *Clostridium difficile* infection (148). Moreover, studies have shown that its success rate is as high as 90%, which means that FMT can restore healthy microbial ecology (149). Therefore, FMT is extended to treat other microbial-related diseases, including UC.

To date, multiple randomized controlled trials (RCTs) have explored the efficacy and safety of FMT in the treatment of UC (Table 2). Among them, 5 RCTs showed that the clinical remission rate of FMT in the treatment of UC was significantly different from that of placebo or autologous fecal transplantation, suggesting that FMT has therapeutic effect on UC (150, 152–155). On the contrast, one study showed that there was no significant difference in the efficacy between FMT and autologous fecal transplantation (156). In addition, another study showed that in UC patients, FMT had worse clinical remission outcomes than 5-ASA, though both had the same clinical response rate (151). These studies seem inconsistent in evaluating the efficacy of FMT. It is worth noting that a meta-analysis involving four FMT-related RCTs finally found that the overall clinical remission rate of FMT group was 28%, which was significantly better than of placebo group, providing some evidence to support the effectiveness of FMT for UC (157). Regarding the different results obtained from these RCTs, it seems biased in relation to specific trials undertaken. For example, experts in a clinical trial found that only one female patient responded to fecal microbiota from male donors, implying that gender may have an impact on the specific efficacy of FMT (151). Second, due to the increased microbial diversity associated with multiple donors, the treatment with multiple donors is more effective than that of individual donor (154, 158). In addition, enema administration is more effective than naso-duodenal tube administration (154). In conclusion, the curative effect of FMT may be influenced by various factors, and more strict and standardized research is needed to explore more standardized strategies for FMT use.

**Table 2:** The characteristics of some randomized controlled trials of FMT for treating UC.

**Study**	**N (FMT/Control)**	**Control**	**Delivery**	**Primary end point**	**Efficacy**	**Safety**
Crothers et al. (150)	12 (6/6)	Sham colonoscopic infusion and sham capsules	Initial colonoscopy then enema and oral maintenance therapy with frozen FMT Capsules.	A mayo score ≤ 2 and an endoscopic sub-score of ≤ 1 at week 12.	FMT group: two subjects, control group: none. (95% CI = 0.38-infinity, *p* = 0.45)	None of the subjects experienced FMT-associated adverse events.
Schierová et al. (151)	16 (8/8)	5-ASA	Enema	A Mayo score ≤ 2, with no subscore > 1 at week 12.	FMT group: 37.5%, control group: 50.0% (*P* = 0.51)	No adverse events were reported during the treatment and 6 weeks after treatment.
Costello et al. (152)	73 (38/35)	Autologous FMT	Initial colonoscopy then enema	A total Mayo score of ≤ 2 (range, 0–12) with an endoscopic Mayo score of ≤ 1 (range, 0–3) at week 8.	FMT group: 32%, control group: 9% (OR, 5.0, 95% CI, 1.2–20.1, *P* = 0.03)	Three serious adverse events in the FMT group and two in the control group with no significant differences.
Sood et al. (153)	61 (31/30)	Saline	Colonoscopic infusion at weeks 0, 8, 16, 24, 32, 40 and 48.	A mayo score ≤ 2, all sub-scores ≤ 1 at week 48.	FMT group: 87.1%, control group: 66.7% (RR 2.2, 95% CI 1.1–4.5; *p* = 0.021)	There were no serious adverse events in FMT group.
Paramsothy et al. (154)	81 (41/40)	Isotonic saline	Initial colonoscopy then enema	A total Mayo score ≤ 2, with all Mayo subscores ≤ 1, and at least a 1-point reduction from baseline in the endoscopy subscore at week 8.	FMT group: 44%, control group: 20% (RR 2.2, 95% CI 1.1–4.5; *p* = 0.021)	Mild adverse events: 78% in the FMT group vs. 83% in the control group with no significant difference. Serious adverse events: two patients in the FMT group vs. one patient in the control group which is not associated with the individual donor or donor batch.
Moayyedi et al. (155)	75 (38/37)	Water	Initial colonoscopy then enema	A full Mayo score <3 and complete healing of the mucosa at flexible sigmoidoscopy at week 7.	FMT group: 24%, control group: 5% (*p* = 0.3).	No difference in serious adverse events between the FMT and placebo groups.
Rossen et al. (156)	48 (23/25)	Autologous FMT	Naso-duodenal tube	A SCCAI score of ≤ 2 in combination with ≥ 1 point improvement on the combined Mayo endoscopic score of the sigmoid and rectum at week 12.	FMT group: 30.4%, control group: 20.0% (*P* = 0.51)	Mild adverse events: 78.3% in the FMT group vs. 64.0% in the control group (*p* = 0.28) Serious adverse events: two patients in the FMT group vs. two patients in the control group.

*FMT, fecal microbiota transplantation; SCCAI, Simple Clinical Colitis Activity Index*.

In terms of safety evaluation, although 4 RCTs reported severe adverse reactions in some patients during treatment, there was no significant statistical difference between FMT and placebo groups (152, 154–156). Furthermore, according to the assessment from principal investigators, the occurrence of severe adverse reactions was not related to FMT treatment. It is worth noting that some experts pointed out that the risk of long-term use of FMT infection transmission is still unclear. In theory, the risk increases with the increase of multiple donors' infusions times, which indicates that the long-term safety of FMT still needs to be studied and evaluated (154).

In addition to adequate evaluating the efficacy and safety, it is also essential to study the effect of FMT interventions on the microbiota of UC patients. Studies have shown that both OUT diversity and Shannon diversity of intestinal microbiota in UC patients after FMT are enhanced (159). Moreover, the abundance of *Bifidobacteriaceae* and *Coriobacteriaceae* increased at the phylum level (160). *Bifidobacteriaceae* has been used as probiotics for the treatment of UC, and *Coriobacteriaceae* plays a significant role in the transformation of bile salts and steroids (161). Furthermore, as an important component of SCFAs, butyrate can regulate intestinal homeostasis. After FMT treatment, the abundance of butyrate-producing bacteria increased significantly and SCFAs recovered (162). In short, FMT can change the microbiota composition of patients, correct microbial dysbiosis, and ultimately exert therapeutic effects for UC by transplanting the microbiota from healthy people to patients. Nevertheless, some problems needed to solved to make FMT a regular treatment strategy for UC. Firstly, a large number of studies are needed to critically evaluate the clinical efficacy and safety of FMT for long-term use. Secondly, the selection of donor sources, whether gender factors and donor diversity need to be considered, and how to avoid the potential infection risks also need to be explored in subsequent studies. Furthermore, there is a lack of standard guidance on specific transplant modalities and treatment periods. In summary, it is undeniable that FMT is a promising treatment for UC. Focusing on FMT research will help its development and maturity.

#### Probiotics, Prebiotics and Synbiotics

Probiotics are active microorganisms that have beneficial effects on the host health at sufficient and accurate doses. A large number of functional foods containing probiotics are safe to eat and have some health effects, suggesting that probiotics are usually safe. At the same time, plenty of studies began to evaluate the therapeutic effects of probiotics on UC. So far, some probiotic species have been proved to have therapeutic potential for UC (Table 3). Among these probiotics, *Escherichia coli* Nissle 1917 and VSL # 3, a probiotic mixture containing *L. paracasei, L. plantarum, L. acidophilus, L. delbrueckii, B. longum, B. breve, B. infantis* and *Streptococcus thermophilus*, are two probiotic products that have been studied more. Studies have shown that VSL # 3 has therapeutic effect on patients with mild to moderate UC, whether for induction therapy or maintenance therapy (174). Moreover, VSL # 3 also has the effect of preventing disease progression (175). The European Society of Nutrition and Metabolism also recommended VSL # 3 and *Escherichia coli* Nissle 1917 as drug for the treatment of mild to moderate UC, suggesting that probiotics do have therapeutic potential for UC (176).

**Table 3:** Probiotics with proven therapeutic potential for UC.

**Probiotic**	**Reference**
*Escherichia coli* Nissle 1917	Kruis et al., Rembacken et al., Kruis et al., (163–165).
*Saccharomyces boulardii*	Guslandi et al., (166).
*Bifidobacteria*	Ishikawa et al., Kato et al., (167, 168).
*Lactobacillus* GG	Zocco et al., (169).
VSL # 3 (a probiotic mixture containing *L. paracasei, L. plantarum, L. acidophilus*, and *L. delbrueckii* subsp *bulgaricus, B. longum, B. breve, B. infantis* and *Streptococcus thermophilus*	Sood et al., Miele et al., Tursi et al., (170, 171).
*Lactobacillus reuteri* ATCC 55730	Oliva et al., (172).
Symprove (a probiotic mixture containing *Lactobacillus rhamnosus* NCIMB 30174, *Lactobacillus plantarum* NCIMB 30173, *Lactobacillus acidophilus* NCIMB 30175 and *Enterococcus faecium* NCIMB 30176)	Bjarnason et al., (173).

The mechanism of probiotics improving UC has not been determined, but different probiotics may play a role in different ways. VSL # 3 inhibits the activation of NF-κB signaling pathway, which is a key factor in the development and persistence of chronic inflammation in UC (177). Another probiotic mixture, Symprove is thought to improve UC by restoring microbial homeostasis, increasing SCFAs production, and restoring epithelial tight junctions (178). In summary, the mechanism of action of probiotics depends on the strains used, and the specificity of some probiotic properties means that often multiple species of probiotics have better therapeutic effects than single probiotics (179). At the same time, a specific probiotic may not be suitable for all UC patients. Therefore, the evaluation of the selection and utilization of probiotics still need to be continued.

Probiotics have been widely studied and gradually applied. Prebiotics are substrates that host probiotic bacteria can selectively utilized and produce health benefits (180). Consequently, prebiotics are used to increase the abundance and activity of probiotics, prolong their lifespan, change the composition of intestinal microbiota, and improve the intestinal barrier function, which is also considered as a mechanism for prebiotics to treat UC (181, 182). Lactulose, inulin, fructooligosaccharide and malt are the most studied prebiotics (183). Although the number of RCTs evaluating the efficacy of prebiotics in the treatment of UC is limited, some preclinical studies and clinical trials have shown their therapeutic potential. Malt is effective for UC to alleviate clinical symptoms and reduce clinical activity index in UC patients (184). Lactulose can improve the quality of life of UC patients (185). Similarly, preclinical studies have found that after treatment with fructans and resveratrol, the abundance of probiotics *Bifidobactrium* and *Lactobacillus* in IBD model rats increase, and starch can induce beneficial changes in the microbiota composition by promoting butyrate production and inhibiting the growth of potentially harmful bacteria (186–188). This evidence supports that prebiotics have the potential to treat UC. However, more well-controlled and high-quality RCTs are urgently needed to further evaluate the therapeutic effects and safety of prebiotics.

Given that both probiotics and prebiotics may have therapeutic potential, the role of synbiotics is also being evaluated. Considering that the probiotic intake can help the host correct the dysbiosis of microbiota, the use of synbiotics, a mixture of probiotics and prebiotics, seems reasonable and prebiotics can also increase the abundance of probiotics and restore the intestinal microbiota (189). Studies have shown that the clinical efficacy of synbiotics in the treatment of UC is significant. The combined application of *bifidobacterial* strains and galactooligosaccharide improved the colonoscopy score and inflammatory markers in UC patients (190). Similarly, a comprehensive meta-analysis showed that synbiotics improved colonoscopy score, clinical activity index and inflammation-related indicators in UC patients (191). In addition, synbiotics can also increase the abundance of probiotics in the intestinal tract of UC patients, and these studies also provide evidence for the treatment of UC by synbiotics.

Since the microbiota is mainly concentrated in the colon, the therapeutic strategies aimed at restoring intestinal microbial homeostasis have a more obvious effect on the colonic microbiota (192). Therefore, probiotics, prebiotics, and synbiotics treatment have more therapeutic value for UC patients than CD patients. In addition, the therapeutic potential of probiotics, prebiotics and synbiotics cannot be ignored in the urgent search for more efficient and safer alternatives to UC. However, the evaluation of their effectiveness and safety is remain inadequate. First, the number of studies on probiotics, prebiotics and synbiotics seems to be decreasing in recent years (Table 4). Second, researchers do not always get positive reactions or conclusions in these RCTs. Some studies have shown that there is no significant different between the efficacy of probiotics and placebo. The underlying reasons may be related to the selection of specific probiotic strains, dosage and the duration of treatment, which also suggests that there is a lack of standard protocol for the use of probiotics. Fortunately, a meta-analysis showed that the use of probiotics increased the risk of side effects compared with placebo, but these symptoms were limited to gastrointestinal reactions and abdominal pain (192). Therefore, the use of probiotics seems to be safer than corticosteroids, thiopurines and other drugs. These drugs always produce severe adverse effects and requires strict follow-up RCTs to re-evaluation.

**Table 4:** The characteristics of some clinical trials of probiotics, prebiotics and synbiotics for treating UC.

**Study**	**N (Treatment/Control)**	**Treatment**	**Species**	**Control**	**Efficacy**	**Safety**
Chen et al. (193)	25 (12/13)	Probiotic	A probiotic product that contained *L. casei* Zhang, *L. plantarum P*-8 and *B. animalis subsp. lactis* V9	Dextrin	The overall remission rate was 91.67% for the probiotic group vs. 69.23% for the placebo group (*P* = 0.034)	-
Bjarnason et al. (173)	81 (40/41)	Probiotic	Symprove (contains *Lactobacillus rhamnosus* NCIMB 30174, *Lactobacillus plantarum* NCIMB 30173, *Lactobacillus acidophilus* NCIMB 30175 and *Enterococcus faecium* NCIMB 30176	Water and flavoring	The calprotectin levels were significantly decreased following 4 weeks in the probiotic group (*p* = 0.011 and 0.001, *t-*test and Wilcoxon's, respectively)	-
Yilmaz et al. (194)	25 (15/10)	Probiotic	Kefir (*Lactobacillus* Bacteria)	-	No statistically significant difference was found between weeks 1 and 2 in patients with UC in terms of abdominal pain, bloating, frequency of stools, defecation consistency, and feeling good.	No adverse events were reported.
Kamarli et al. (183)	36 (18/18)	Synbiotic	A symbiotic which concluded six probiotics: *Enterococcus faecium, Lactobacillus plantarum, Streptococcus thermophilus, Bifidobacterium lactis, Lactobacillus acidophilus, Bifidobacterium longum* and fructooligosaccharide.	Placebo product which has the same taste and appearance	The change in the CRP and sedimentation values had a statistically significant decrease in the synbiotic group (*P* = 0.003). The improvement in the clinical activity was significantly higher in the synbiotic group (*p* < 0.05).	-
Yoshimatsu et al. (195)	46 (23/23)	Probiotic	A tablet contains *Streptococcus faecalis* T-110, *Clostridium butyricum* TO-A and *Bacillus mesentericus* TO-A	A placebo tablet which contains starch	The relapse rates in the treatment and placebo groups were 0.0% vs. 17.4% at months (*p* = 0.036). At 12 months, the remission rate was 69.5% in the treatment group and 56.6% in the placebo group (*p* = 0.248).	-
Matsuoka et al. (196)	192 (97/95)	Probiotic	Mil–Mil (a fermented milk product containing *B. breve* strain Yakult and *Lactobacillus acidophilus*	-	Relapse-free survival was not significantly different between the treatment and placebo groups (*P* = 0.643)	Three mild adverse events occurred which could not be ruled out whether is associated with the probiotic.

#### The Application of TCM in Treating UC

##### The Potential Effect of TCM on Intestinal Microbiota

Due to the complex active ingredients, unclear pharmacological effects, and low oral availability of Chinese medicinals, it is difficult to fully clarify its effective mechanism, which is a great challenge to TCM. However, with intestinal microbiota becoming an emerging field to understand the occurrence and development of diseases in recent years, researchers begin to pay attention to TCM. On this basis, it is found that the pharmacological effects of TCM are related to the intestinal microbiota (12).

Most medicinals are oral. Low oral availability compounds from medicinals can reach the colon, which is the most concentrated part of the intestinal microbiota (197). Medicinals will inevitably be exposed to intestinal microbiota, providing the necessary conditions to affect the intestinal microbiota (198). After TCM compounds enter the intestinal tract, the composition and metabolism of the intestinal microbiota are regulated directly and indirectly (12).

The intestinal microbiota participates in the transformation of TCM compounds by expressing corresponding biological enzymes to activate, inactivate, or reactivate TCM compounds (199). Berberine is the primary pharmacological component of *huáng lián* (the dried rhizome of *Coptis chinensis* Franch.), with low oral availability and maximum blood concentration in human body. Interestingly, the berberine ethanol extracts significantly reduced the abundance of *Firmicutes* and *Bacteroides* in the feces of HFD mice (200). Meanwhile, the diversity and total number of intestinal microbiotas treated with berberine were also significantly reduced (201). The use of TCM is often not only an herb blindly but also a combination of medicinals with different effects through compatibility. *Huáng Q*í*n Tāng* (Scutellaria Decoction), widely used in the treatment of gastrointestinal diseases, has been found that its improvement effect on UC is related to intestinal microbiota regulation (202). In short, some medicinals with low oral availability can exert sound effects in the treatment of various diseases, which is closely related to intestinal microbiota.

##### The Preclinical Study of Application TCM in Animal Models of UC

TCM is characterized by abundant resources and clinical safety. Numbers of researchers have invested in TCM for treating UC and made some progress (9, 10). The research on animal experiments is essential in discovering suitable therapeutic drugs and studying their possible mechanisms. Consequently, recent animal studies have shown that TCM may have a potential to be applied to UC patients. According to the different TCM selected by the researchers in animal experiments, we categorized the investigated medicinals into three parts: compound extracted from Chinese medicinals and single medicinal, couplet medicinal, and Chinese medicinal formula.

*Compound Extracted From Chinese Medicinals and Single Medicinal*. Although a single medicinal is rarely used in clinical practice, relative studies can identify its unilateral effectiveness in UC. The selection of specific medicinals, animal models, and study results of these experiments are shown in Table 5. Under the guidance of TCM theory, the particular efficacy of TCM and pharmacological research will become the essential reference for researchers to choose TCM. On this basis, choosing appropriate TCM for animal experiments is more likely to find medicinals with obvious therapeutic effects on UC.

**Table 5:** Application of compound extracted from Chinese medicinal (or single medicinals) in animal models of UC.

**Medicinals**	**Compounds**	**Animals**	**Experimental methods**	**Results (symptoms, cytokines and pathways)**	**Results(Intestinal Microbiota)**	**References**
*qīng dài* (the dried processed product of leaf or stem and leaf of *Strobilanthes cusia* (Nees) Kuntze)	Indirubin Indigo	BALB/c mice SD rats	DSS	It inhibited the loss of bodyweight, reversed the elevation of DAI store, alleviated crypt distortion and mucosal injury, and reduced inflammatory cell infiltration in the colon mucosa. TNF-α, IFN-γ, IL-2, MPO were decreased. IL-4, IL-10 were increased. Few CD4+ T cells were observed in colon tissues. The activation of NF-κB signaling was inhibited.	α-diversity was increased. At the phylum level, *Firmicutes* and *Actinobacteria* were increased, *Bacteroidetes* was decreased. At the family level, the abundance of *bifidobacteriaceae* and *Ruminococcaceae* was increased.	(203, 204)
*huáng qín* (the dried root of *Scutellaria baicalensis* Georgi)	Oroxindin	C57BL/6 mice	DSS	Oroxindin suppressed massive macrophages infiltration and attenuated pathological changes in colonic tissue. The expression of IL-1β, IL-18, caspase-1 and p-p65 were decreased, it suggested that Oroxindin inhibited NLRP3 inflammasome formation and NF-κB activation.	-	(205)
*dà xuè téng* (the dried vine stems of *Sargentodoxa cuneata* (Oliv.) Rehder & E.H.Wilson)	Liriodendrin	BALB/c mice	DSS	Liriodendrin improved DAI, colon length and histological damage in colon of mice. MPO, IL-6, TNF-α, and IL-1β were reduced. It also suppressed the activation of Akt and NF-κB pathways and up-regulated the expression of Erβ.	-	(10)
*chuān xin lián* (the dried above-ground part of *Andrographis paniculata* (Burm.f.) Nees)	3,14,19-triacetyl andrographolide	BALB/c mice	DSS	It reduced body weight loss, colon length shortening, colon weight, the spleen index, and DAI store, and alleviated histological damage in the colon. MPO, TNF-α, and IL-6 were decreased. It could inhibit the activation of NF-κB and MAPK pathways.	-	(206)
*qiàn căo* (the dried root and rhizome of *Rubia cordifolia* L.)	Mollugin	C57BL/6 mice	DSS	Mollugin decreased the DAI scores and histological score. IL-1β and TNF-α were decreased. The level of TLR4 was decreased.	-	(207)
*mù xiāng* (the dried root of *Aucklandia costus* Falc.)	-	SD rats	TNBS	*Mù xiāng* ameliorated stomachache, diarrhea and hematochezia of rats. Body weight was increased. IL-1β, TNF-α, caspase-3, BAD were decreased. IL-6, IL-10, p53 and Bcl-2 were upregulated. The activity level of PI3K and Akt was increased.	-	(208)
*shí hú* (the fresh or dried stem of *Dendrobium nobile* Lindl.)	Polysaccharides	BALB/C mice	DSS	Polysaccharides of *shí hú* could improve clinical signs and symptoms, decrease mortality, alleviate colonic pathological damage. IL-1β, IL-6, IL-18, TNF-α, and IFN-γ were decreased. IL-10 was increased. It could also suppress the activation of NLRP3 inflammasome and β-arrestin1.	-	(209)

*DAI, disease activity index; TNF-α, tumor necrosis factor-α; IL, interleukin; MPO, myeloperoxidase; NF-κB, nuclear factor κB; Akt, protein kinase B; TLR, Toll-like receptor; Bcl-2, apoptosis regulator Bcl-2; BAD, Bcl2-associated agonist of cell death; PI3K, phosphatidylinositol 3-kinase; IFN-γ, interferon-gamma*.

*Qīng dài* (the dried processed product of leaf or stem and leaf of *Strobilanthes cusia* (Nees) Kuntze), *qiàn c*ă*o* (the dried root and rhizome of *Rubia cordifolia* L.) and *huáng q*í*n* (the dried root of *Scutellaria baicalensis* Georgi) have the effect of clearing heat, and its pharmacological effects with anti-inflammatory and antibacterial have also been proved (210, 211). In animal experiments, it was found that these medicinals have an improvement effect on the general symptoms such as diarrhea, bloody stool of animals and colon inflammation in experimental colitis. At the same time, the disease activity index (DAI) score and histopathological score also showed a downward trend.

The release of pro-inflammatory cytokines plays an essential role during the development of UC (212). Compared with the model group, the expressions of pro-inflammatory cytokines such as IL-1β, IL-2, IL-6, IL-18, TNF-α and interferon-gamma (IFN-γ) decreased obviously after administration. The levels of IL-4 and IL-10, considered to be anti-inflammatory cytokines, increased after administration. The use of medicinals also reduced the levels of pro-inflammatory cytokines in animals to alleviate experimental colitis.

Abnormal activation of signaling pathways is often the key to induce inflammation. The activation of NF-κB and mitogen-activated protein kinase (MAPK) signaling pathways have been found to be important factors leading to the occurrence and development of UC (213). Similarly, medicinals also inhibit the signaling pathways such as NF-κB and MAPK in animal experiments. The phosphorylation of protein kinase B (Akt) was increased in mice after the induction of DSS, while *dà xuè téng* (the dried stem of *Sargentodoxa cuneata* (Oliv.) Rehder & E.H.Wilson) reversed this alteration (10).

*Chuān xīn lián* (the dried above-ground part of *Andrographis paniculata* (Burm.f.) Nees) not only down-regulates the expression of NF-κB p65 and p-IκBα, inhibits the activation of NF-κB, but also down-regulates the phosphorylation of MAPK subfamily-related kinases (extracellular signal-regulated kinase, p38mapk, and c-Jun amino-terminal kinase), thereby inhibiting the activation of MAPK signaling pathway to alleviate DSS-induced UC (206). Besides, the application of *qīng dài* (203, 204), *huáng q*í*n* (205), and *sh*í *hú* (the fresh or dried stem of *Dendrobium nobile* Lindl.) (209) also showed an inhibitory effect on the NF-κB signaling pathway. Briefly, the inhibition of key signaling pathways can improve or relieve UC, which also offers a theoretical basis for the efficacy of TCM.

The pharmacological effects of medicinals in the human body lie in their interaction with the intestinal microbiota. The abnormal host immune response caused by the dysbiosis of intestinal microbiota is considered to be the critical mechanism for UC (46), so the essential role of intestinal microbiota in the pathogenesis of UC has been attracted more and more attention (214). Of course, we also found that some scholars have focused their researches on intestinal microbiota with TCM in treating UC. DSS-induced rats have a dysbiosis of the intestinal microbiota, including a decrease in the diversity of the microbiota, a reduction in the abundance of *Firmicutes*, and an increase in the abundance of *Bacteroidetes*. After the treatment of *qing dài*, the diversity of the rat's bacteria and the balance between the two microbiotas were restored. It is considered that *qing dài* modulates its immune response by changing the intestinal microbiota, finally reduce DSS-induced colitis (203).

Many animal experiments conducted on a single medicinal show that TCM can treat experimental colitis by inhibiting the activation of related inflammatory signal pathways and the release of pro-inflammatory cytokines. Besides, some researchers have noticed that it has a therapeutic effect on experimental colitis by improving the intestinal microbiota, indicating that the potential of TCM in the treatment of UC has been proved.

*Couplet Medicinal*. Couplet medicinal is a combination of two medicinal that enhances curative effect or reduces toxicity according to the principle of TCM treatment. It is also the basic unit of Chinese medicinal formula (215). Researches on couplet medicinals help to dispel the interactions between medicinals and explain the mechanism behind them. The corresponding couplet medicinals are shown in Table 6.

**Table 6:** Application of couplet medicinals in animal models of UC.

**Medicinals**	**Compounds**	**Animals**	**Experimental methods**	**Results (symptoms, cytokines and pathways)**	**Results(Intestinal Microbiota)**	**References**
*huáng qín* (the dried root of *Scutellaria baicalensis* Georgi) and *huáng lián* (the dried rhizome of *Coptis chinensis* Franch.)	Baicalin and berberine hybrid compound	BALB/c mice	DSS	It ameliorated the disease symptoms and prevented the colon damage of mice. Expression of MPO, IL-1β, TNF-α and IL-6 were decreased. Level of SOD was increased.	-	(215)
*dăng shēn* (the dried root of *Codonopsis pilosula* (Franch.) Nannf.) and *huáng qí* (the dired root of *Astragalus mongholicus* Bunge)	Total polysaccharides of *huáng qí* and total polysaccharides of *dăng shēn*	C57BL/6 mice	DSS	It alleviated weight loss and DAI score of mice. The level of MLN coefficient, MDA, IL-1β, TNF-α, IL-6 were decreased. In contract, SOD, IL-10, IL-22 were increased.	The level of *Firmicutes* and *Proteobacteria* was down-regulated. *Bacteroidetes* was up-regulated. Also, production of butyrate and overall microbiota structure were improved.	(216)
*fù zi* (the processed lateral root of *Aconitum carmichaelii* Debeaux) and *gān jiāng* (the dried rhizome of *Zingiber officinale* Roscoe)	-	C57BL/6 mice	DSS	*Fù zi* and *gān jiāng* significantly ameliorated the clinical symptoms of body weight loss, colonic shortening, increased DAI and splenomegaly, as well as histological scores of UC mice. IFN-γ, TNF-α, IL-1β, IL-6, IL-10 and IL-17A were suppressed. The levels of MPO, iNOS and COX-2 mRNA were suppressed too. The activation of MAPK, NF-κB and STAT3 signaling pathways were inhibited.	-	(217)

*MPO, myeloperoxidase; IL, interleukin; TNF-α, tumor necrosis factor-α; SOD, superoxide dismutase; MLN, mesenteric lymph node; DAI, disease activity index; IFN-γ, interferon-gamma; iNOS, inducible nitric oxide synthase; COX-2, cyclooxygenase-2; MAPK, mitogen-activated protein kinase; NF-κB, nuclear factor κB*.

*Huáng q*í*n* and *huáng lián* is a combination commonly used in the treatment of intestinal diseases. Baicalin and berberine are two main chemical constituents extracted from these medicinals. And the baicalin-berberine complex was found in *Huáng q*í*n-huáng lián* decoction (218). After administration of baicalin-berberine hybrid compound to DSS-induced mice, the myeloperoxidase (MPO) activity in colon tissue and the expression of pro-inflammatory cytokines (TNF-α, IL-1β, and IL-6) were inhibited, and the development of inflammation was prevented. Besides, the therapeutic effect of baicalin-berberine complex was significantly better than that of baicalin and berberine (215).

Another couplet medicinal, *d*ă*ng shēn* (the dried root of *Codonopsis pilosula* (Franch.) Nannf.) and *huáng q*í (the dired root of *Astragalus mongholicus* Bunge), is also commonly used in the Chinese medicinal formula for treating UC. The polysaccharides in this combination improved the symptoms of experimental colitis mice and alleviated the intestinal mucosa injury. At the same time, the diversity of intestinal microbiota recovered with the increase of *Bacteroidetes* abundance as well as the decrease of *Firmicutes* and *Proteobacteria* (216). Moreover, the therapeutic effects of the polysaccharides of *d*ă*ng shēn* and *huáng q*í on UC are better than that of a single polysaccharide.

In addition to enhancing the efficacy of medicinals, the couplet medicinals can also reduce the toxicity of TCM. *fù zi* (the processed lateral root of *Aconitum carmichaelii* Debeaux) and *gān jiāng* (the dried rhizome of *Zingiber officinale* Roscoe) are commonly used to treat diarrhea (219). However, hypaconitine, mesaconitine, and aconitine are the main toxic chemical composition in *fù zi* (220). After the co-use of *fù zi* and *gān jiāng*, the dissolution of hypaconitine and mesaconitine significantly reduced, indicating that the compatibility is beneficial to reduce the toxicity of *fù zi*. Meanwhile, the combination can reduce the release of pro-inflammatory cytokines by inhibiting the MAPK, NF-κB, and signal transducer and activator of transcription 3 (STAT3) signaling pathways in the DSS-induced colitis (217).

The use of couplet medicinals can often improve the efficacy of TCM for experimental colitis. Besides, the reasonable compatibility of some toxic medicinals can also reduce its harmful effects, which also provides strong evidence for the treatment of UC by TCM.

*Chinese Medicinal Formula*. Under the guidance of the TCM theory, the Chinese medicinal formula is to combine different single medicinal or couplet medicinals into a complete formula with corresponding effects (221). The outstanding multi-target synergistic effect of Chinese medicinal formula provides a certain basis for its rationality (222). The selection of specific formulas and study results are shown in Table 7. Each Chinese medicinal formula usually comprises different medicinals, but we found that *huáng q*í*n, huáng lián, huáng q*í*, d*ă*ng shēn* and *qīng dài* were widely used in these selected formulas in animal experiments. In the DSS-induced colitis in rats or mice, the general symptoms of the animals were improved compared with the model groups. The DAI score and histopathological score were decreased. Meanwhile, the decreased expression of various pro-inflammatory cytokines also suggests the effect of Chinese medicinal formulas in reducing UC.

**Table 7:** Application of Chinese medicinal formulas in animal models of UC.

**Formulas**	**Medicinals**	**Animals**	**Experimental methods**	**Results (symptoms, cytokines and pathways)**	**Results(Intestinal Microbiota)**	**References**
*Bàn Xià Xiè Xīn Tang* (Pinellia Heart-Draining Decoction)	*bàn xià* (the dried tuber of *Pinellia ternata* (Thunb.) Makino), *huáng qín* (the dried root of *Scutellaria baicalensis* Georgi), *huáng lián* (the dried rhizome of *Coptis chinensis* Franch.), *gān jiāng* (the dried rhizome of *Zingiber officinale* Roscoe), *rén shēn* (the dried root and rhizome of *Panax ginseng* C.A.Mey.), *gān căo* (the dried root and rhizome of *Glycyrrhiza uralensis* Fisch. ex DC.), *dà zăo* (the dried ripe fruit of *Ziziphus jujuba* Mill.)	C57BL/6 mice	DSS	It ameliorates body weight loss, DAI and histology score. TNF-α, IL-1β, IL-17, IL-23, COX-2, p-p65, MPO and 8-Oxoguanine were decreased. IL-10, SOD activity and Nrf2 expression were elevated.	-	(221)
*Dà Huáng Mŭ Dān Tāng* (Rhubarb and peony bark Decoction)	*dà huáng* (the dried root and rhizome of *Rheum palmatum* L.), *mŭ dān pí* (the dried velamen of *Paeonia × suffruticosa* Andrews), *táo rén* (the dried ripe seed of *Prunus persica* (L.) Batsch), *máng xiāo* (Natrii Sulfas), *dōng guā zi* (the dried ripe seed of *Benincasa hispida* (Thunb.) Cogn.)	C57BL/6 mice	DSS	*Dà Huáng Mu Dān Tāng* rescued the inflammation-related reduction of colon length, ameliorated body weight loss and damaged tissue of mice. The level of IL-6, TNF-α, IFN-γ, IL-10, IL-17A, IL-21, IL-22 in colon was decreased. The Th17/Treg balance was restored.	α-diversity of gut microbiota was restored. Abundance of *Firmicutes* and *Actinobacteria* was increased. *Proteobacteria* was decreased. The content of SCFA in intestinal tract was restored.	(223)
*Shēn Líng Bái Zhú Săn* (Ginseng, Poria and Atractylodes Macrocephalae Powder)	*rén shēn, fú líng* (the dried sclerotia of *Poria cocos* (Schw.) Wolf), *bái zhú* (the dried rhizome of *Atractylodes macrocephala* Koidz.), *shān yào* (the dried rhizome of *Dioscorea oppositifolia* L.*), bái biăn dòu* (the dried ripe seed of *Lablab purpureus subsp. purpureus), lián zi* (the dried ripe seed of *Nelumbo nucifera* Gaertn.), *yì yi rén* (the dried ripe seed kernel of *Coix lacryma-jobi var. ma-yuen* (Rom.Caill.) Stapf), *shā rén* (the dried ripe fruit of *Wurfbainia villosa* (Lour.) Skornick. & A.D.Poulsen), *jié gĕng* (the dried root of *Platycodon grandiflorus* (Jacq.) A.DC.), *gān căo*	C57BL/6 mice	DSS	It could increase body weight and colon length of UC mice, decrease the DAI score and improve colonic injury. The production of IL-1β, IL-18, and TNF-α was decreased. It also inhibited the MAPK and NF-kB signaling pathways.	-	(224)
*Jiàn Pí Qīng Cháng Tāng* (Spleen-Fortifying and Intestine-Clearing Decoction)	*huáng qí* (the dired root of *Astragalus mongholicus* Bunge), *dăng shēn* (the dried root of *Codonopsis pilosula* (Franch.) Nannf.), *mă chĭ xiàn* (the dried above-ground part of *Portulaca oleracea* L.), *dì yú* (the dried root of *Sanguisorba officinalis* L.), *sān qi* (the dried root and rhizome of *Panax notoginseng* (Burkill) F.H.Chen*), bái jí* (the dried tuber of *Bletilla striata* (Thunb.) Rchb.f.*), mù xiāng* (the dried root of *Aucklandia costus* Falc.), *huáng lián, gān căo*	C57BL/6 mice	DSS	*Jiàn Pí Qīng Cháng Tāng* increased body weight and colon length of UC mice and decreased DAI score. TNF-α and IL-1β were decreased. It could inhibit the NF-κB/HIF-1α signaling pathway.	-	(222)
*Píng Wèi Săn* (Stomach-Calming Powder)	*cāng zhú* (the dried rhizome of *Atractylodes lancea* (Thunb.) DC.), *hòu pò* (the dried bark of *Magnolia officinalis* Rehder & E.H.Wilson), *chén pí* (the dried ripe pericarp of *Citrus reticulata* Blanco), *gān căo, shēng jiāng* (the fresh rhizome of *Zingiber officinale* Roscoe), *dà zăo*	C57BL/6 mice	DSS	*Píng Wèi Săn* improved body weight, bloody feces and diarrhea of UC mice. MPO, IL-17A and IFN-γ mRNA levels were decreased.	The abundance of microbiota in mice was restored. In the phylum level, *Firmicutes* was increased and *Bacteroidetes* was decreased. Besides, the abundance of *Lactobacillus* was restored. The level of LPS in serum was reduced.	(225)
*Qīng Cháng Huà Shi Tāng* (Intestine-Clearing and Dampness-Removing Decoction)	*huáng qí, bái sháo* (the dried root of *Paeonia lactiflora* Pall.), *bái tóu wēng* (the dried root of *Pulsatilla chinensis* (Bunge) Regel), *bái zhĭ* (the dried root of *Angelica dahurica* (Hoffm.) Benth. & Hook.f. ex Franch. & Sav.), *huáng qín, dì yú*	C57BL/6 mice	DSS	It could improve weight loss, diarrhea, and rectal bleeding of DSS-treated mice. Expression of IL-1β, IL-6, TNF-α, NLRP3, caspase1, and IL-18 were decreased, Muc2 and Reg3γ were increased.	α-diversity of intestinal microbiota was improved. The level of *Firmicutes* was increased. In contrast, the level of *Bacteroidetes* was decreased.	(226)
*Qīng Cháng Wēn Zhōng Tāng* (Intestine-Clearing and Center-Warming Decoction)	*huáng lián, páo jiāng* (the processed product of root of Zingiber officinale Roscoe), *kŭ shēn* (the dried root of *Sophora flavescens* Aiton), *qing dài, dìyútàn* (the processed product of root of *Sanguisorba officinalis* L.), *mù xiāng, sān qī, gān căo*	SD rats	DSS	*Qīng Cháng Wēn Zhōng Tāng* improved weight loss, DAI score, and histological score of DSS-induced rats. Expression of IP10, CXCR3 and NF-κB p65 were decreased.	-	(227)
*Sān Huáng Shú Ài Tāng* (Scutellaria, Coptis, phellodendron bark and Mugwort Decoction)	*huáng lián, huáng qín, huáng băi* (the dried bark of *Phellodendron chinense* C.K.Schneid.), à*i yè (*the dried leaf of *Artemisia argyi* H.Lév. & Vaniot)	BALB/C mice	DSS	*Sān Huáng Shú Ài Tāng* restored weight loss, colon length of UC mice. DAI score wad decreased. IL-1β, IL-6, TNF-α, P-p65, MPO and MDA were reduced.	Species of intestinal flora were restored. The abundance of *Lactobacillus sp*. was increased.	(228)
*Huáng Lián Jiě Dú Tāng* (Coptis Toxin-Resolving Decoction)	*huáng lián, huáng qín, huáng băi, zhi zi* (the dried ripe fruit of *Gardenia jasminoides* J.Ellis)	BABL/c mice	DSS	*Huáng Lián Jiě Dú Tāng* decreased DAI score, inhibited weight loss and colon shortening of UC mice. IL-1β and TNF-α were reduced, IL-10 was increased. It could suppress NF-κB signaling pathway, activating Nrf2 signaling pathway, and enhancing intestinal barrier function.	-	(229)
*Sūqīng Wán*	*jin yín huā* (the dried bud of flower of *Lonicera japonica* Thunb.), *lián qiào* (the dried fruit of *Forsythia suspensa* (Thunb.) Vahl), *pú gōng yīng* (the dried whole grass of *Taraxacum mongolicum* Hand. -Mazz.), *dì yú, tiān huā fen* (the dried root of *Trichosanthes kirilowii* Maxim.), *bái zhǐ, shēng dì* (the fresh or dried tuberous root of *Rehmannia glutinosa* (Gaertn.) DC.), *shēng má* (the dried rhizome of *Actaea cimicifuga* L.), *huáng qí, dāng guī* (the dried root of *Angelica sinensis* (Oliv.) Diels), *ji nèi jīn* (Endothelium Corneum Gigeriae Galli), *xuán shēn* (the dried root of *Scrophularia ningpoensis* Hemsl.), *gān căo*	Kunming mice	DSS	*Sūqīng Wán* decreased DAI score and attenuated symptoms of UC mice. TNF-α, IL-1β, IL-6, MPO, and MDA were reduced.IL-4 and IL-10 were increased.	-	(230)
*Bā wèi Xi lèi Sǎn*	*xi guā shuāng* (Mirabilitum Praeparatum), *hán shuĭ shí* (Glauberitum), *niú huáng* (Calculus Bovis), *zhēn zhū* (Margarita), *péng shā* (Borax), *bing piàn* (Borneolum Syntheticum), *náo shā* (Sal Ammoniacum), *qīng dài*	C57BL/6 mice	DSS	*Bā wèi Xi lèi Sǎn* improved body weight loss and colon length of DSS-treated mice. The expression level of Th17-related cytokines IL-17A/F and IL-22 was significantly reduced, resulting in the restoration of Th17/Treg balance.	The level of *Lactobacillus* was improved.	(46)

*DAI, disease activity index; TNF-α, tumor necrosis factor-α; IL, interleukin; COX-2, cyclooxygenase-2; MPO, myeloperoxidase. SOD, superoxide dismutase; Nrf2, Nuclear factor E2-related factor 2; IFN-γ, interferon-gamma; MAPK, mitogen-activated protein kinase; NF-κB, nuclear factor κB; HIF-1α, Hypoxia-inducible factor-1α; Reg3γ, regenerating gene 3γ; IP10, Interferon gamma-induced protein 10; CXCR3, Chemokine (cys-x-cys motif) receptor 3; MDA, malondialdehyde*.

The diversity of the constituent medicinals of a Chinese medicinal formula determines the difference in its mechanism of action. *Bàn Xià Xiè Xīn Tang* (Pinellia Heart-Draining Decoction) (221), *Shēn L*í*ng Bái Zhú S*ă*n* (Ginseng, Poria and Atractylodes Macrocephalae Powder) (224), *Jiàn P*í *Qīng Cháng Tāng* (Spleen-Fortifying and Intestine-Clearing Decoction) (222), *Sān Huáng Shú Ài Tāng* (Scutellaria, Coptis, Phellodendron bark and Mugwort Decoction) (228) and *Huáng Lián Jiĕ Dú Tāng* (Coptis Toxin-Resolving Decoction) (229) can inhibit the signal of NF-κB Pathways to reduce colon inflammation. *Qīng Cháng Wēn Zhong Tāng* (Intestine-Clearing and Center-WarmingDecoction) is related to the inflammation mediated by the interferon gamma-induced protein 10(IP10)/Chemokine (cys-x-cys motif) receptor 3 (CXCR3) axis (208). Moreover, *Suqīng Wán* is thought to be involved in the up-regulation of anti-inflammatory cytokines and down-regulation of pro-inflammatory and oxidative factors (230).

The effect of Chinese medicinal formula on intestinal microbiota has also been confirmed. The microbial dysbiosis in UC patients includes the decreased diversity and microbiota composition disorder (231). *Dà Huáng Mŭ Dān Tāng* (Rhubarb and peony bark Decoction) (223), *P*í*ng Wèi S*ă*n* (Stomach-Calming Powder) (225), *Qing Cháng Huà Shī Tāng* (Intestine-Clearing and Dampness-Removing Decoction) (226), *Sān Huáng Shú Ài Tāng* (228) and *Bā wèi Xī lèi Sǎn* (232) all could restore the diversity of the intestinal microbiota in DSS-treated mice. Meanwhile, it was found that the intestinal bacteria in DSS-treated mice also showed a recovery after administration: the abundance of *Firmicutes* increased, the amount of *Bacteroides* and *Proteobacteria* decreased, and the amount of *lactobacillus* increased.

Intestinal bacteria are responsible for the biotransformation of bile acids and the production of SCFAs in the intestinal lumen. The dysbiosis of intestinal bacteria will be secondary to the imbalance of the microbiota metabolites and products in the development of UC (5). Interestingly, *Qīng Cháng Huà Shi Tāng* restores the balance between BAs metabolism and SCFAs production (226).

Single medicinal, couplet medicinal and Chinese medicinal formula all can play an obvious therapeutic effect on UC through animal experiments, which proves the effectiveness of TCM. The inhibition of inflammatory signaling pathways, the reduction of pro-inflammatory cytokines expression, and the restoration of intestinal microbiota disorders also indicate the critical mechanism of TCM in treating UC.

##### The Clinical Application of TCM in Treating UC

The most common clinical manifestations of UC are mucus, bloody stool, and diarrhea (1), which correspond to diseases such as “dysentery,” “chronic dysentery,” and “intestinal diarrhea” in TCM (233). According to the consensus opinion of the TCM diagnosis and treatment of UC issued by the Spleen and Stomach Diseases Branch of the China Association of Chinese Medicine in 2017, it is believed that TCM should diagnose and treat UC with “chronic dysentery” because that UC also has the characteristics of recurring attacks and difficult to be cured.

According to the principle of pattern differentiation and treatment and the latest consensus opinion, UC can be divided into seven syndromes characterized by large intestinal damp-heat, spleen deficiency and damp accumulation, intense heat toxin, cold and heat in complexity, liver constraint and spleen deficiency, spleen-kidney yang deficiency, and yin-blood depletion. In clinical practice, intestinal damp-heat spleen deficiency and damp accumulation are the two most common syndromes in UC patients (234). Patients with intestinal damp-heat syndrome can use the prescription *Sháo Yào Tāng* (Peony Decoction), while patients with spleen deficiency and damp accumulation syndrome can use *Shēn L*í*ng Bái Zhú S*ă*n* for treatment. Interestingly, syndrome differentiation determines that the use of TCM is individualized. Doctors always prescribe the appropriate formulas or medicinals according to the symptoms and signs of patients. When conducting animal experiments to evaluate the therapeutic effects of TCM, researchers often choose medicinals and formulas with afficacy of clearing heat, strengthening the spleen, and drying dampness. Moreover, the main chemical constituents of these medicinals have been proven to have antibacterial and anti-inflammatory effects similar to antibiotics. Through the analysis of medication rules of UC in TCM from 2000 to 2020, it was found that medicinals such as *huáng lián, ku shēn* (the dried root of *Sophora flavescens* Aiton), *huáng q*í*n, mù xiāng* (the dried root of *Aucklandia costus* Falc.), and *bái zhú* (the dried rhizome of *Atractylodes macrocephala* Koidz.) are used frequently (235).

The heat-clearing medicinals are the most widely used for classification based on efficacy. Similar results were obtained by analyzing the formulas related to the treatment of UC in the Chinese patent database. Among these formulas, *huáng lián* is the most frequently used medicinal (236). Notably, the formula *Sháo Yào Tāng* and *Shēn L*í*ng Bái Zhú S*ă*n* mentioned in the consensus opinion also contain medicinals such as *huáng lián, huáng q*í*n*, and *bái zhú*. Thus, it can be found that medicinals with heat-clearing effects are critical among the specific medications currently used in TCM to treat UC.

TCM has been widely used in the clinical treatment of UC in China. In the retrospective study on 247 UC patients, TCM was chosen in mild to moderate UC patients and used as an adjuvant treatment for severe UC. It was finally found that TCM treatment is indeed effective (237). At the same time, the application of TCM showed the same therapeutic effect as that of mesalazine with fewer adverse reactions (238, 239). Another medicinal, *qing dài*, was found to alleviate moderate active UC significantly (240). The results of a randomized controlled trial on the treatment of UC by *Jiàn P*í *Qing Cháng Tāng* showed that it could improve the clinical symptoms of patients with mild to moderate UC and improve the quality of life (241). Undoubtedly, these studies provide more evidence for the effectiveness of TCM in treating UC. However, sufficient evidence-based medical evidence is needed to support its effectiveness and safety for long-term use. Moreover, given the typically low quality of existing TCM-related randomized controlled trials, there remains an urgent need for more rigorous randomized controlled trials to offer high-quality evidence for the appropriate application of various TCM.

##### Potential and Deficiency of TCM

In a series of animal experiments, alleviation of animal symptoms, reducing inflammation, and decreasing expression of pro-inflammatory cytokines all suggest the therapeutic potential of TCM on experimental colitis. Most researchers focus on the critical inflammatory signal pathways affected by TCM, including NF-κB and MAPK pathways, and study the mechanism of action. Interestingly, some medicinals have also shown significant regulatory effects on the intestinal microbiota, including increasing the diversity and changing the composition of the microbiota. Therefore, TCM can restore the intestinal microbial homeostasis, reduce the damage of pathogenic bacteria and opportunistic pathogens to the epithelial barrier, inhibit the abnormal activation of key inflammatory signaling pathways, and ultimately reduce the abnormal immune response of the host, which may be the key mechanism of TCM in treating UC.

Moreover, medicinals with heat-clearing effects prioritized treating UC in the animal experiment stage and specific clinical practice. Heat-clearing medicinals have cold or cool properties in nature according to the theory of TCM. Pharmacological studies have shown that they have antibacterial and anti-inflammatory effects. Notably, excessive and long-term use of heat-clearing herbs can damage the “yang” of the stomach and spleen, resulting in abdominal pain, and diarrhea and other gastrointestinal symptoms. To a certain extent, it is still unclear whether the irrational use of heat-clearing drugs in UC patients will further destroy the intestinal microbial homeostasis and eventually lead to the aggravation of UC. Consequently, more preclinical studies and evidence-based clinical studies are needed to support whether heat-clearing medicinals as critical drugs for treating UC and establishing reasonable use specifications to obtain better clinical benefits.

## Conclusion

According to current evidence, the occurrence of UC is associated with multiple risk factors such as heredity, immunity and microorganisms. It is worth noting that more and more studies have pointed out that the intestinal microbiota may play a key role in the development of UC. What's more, the microbial disorders are also related to abnormal immune system. Therefore, further study of intestinal microbiota may be a prospective direction to elucidate the pathogenesis of UC. Similarly, it is found that treatments aimed at correcting the imbalance and restoring the homeostasis of intestinal microbiota may be an essential strategy for the treatment of UC. In many animal studies and clinical practices, FMT, probiotics, prebiotics and synbiotics treatment have shown great therapeutic potential for UC. At the same time, more researches are needed to support their efficacy and safety, and to explore more standardized approaches in their application. Likewise, TCM has obvious advantages in the treatment of UC, and it has systematic and comprehensive advantages in regulating intestinal microbiota and human body, and has potential and practical value in the treatment of UC. In addition, animal models are basic tools for studying the pathogenesis of diseases and exploring therapeutic strategies. In the most commonly used models, the DSS-induced UC model is closer to the occurrence and development of UC, but the role of microbiota in it seems unclear or even controversial. In conclusion, it is necessary to study the roles of microbiota in the pathogenesis of UC and the improvement of UC by microbial therapy.

## Author Contributions

YH, ZY, and MW proposed the idea. YH and ZY searched the literature. YH, ZY, MW, YS, KQ, and YX reviewed and summarized the literature. ZH, MY, FL, and QY modify the manuscript. LL was contributed to the figures and tables. All authors read and approved the final manuscript.

## Funding

This work was supported by the National Natural Science Foundation of China (no. 81973742), the Science and Technology Department of Sichuan Province (nos. 2020YJ0383 and 20YYJC0910), and the Sichuan Youth Science and Technology Innovation Research Team Project (no. 2020JDTD0022).

## Conflict of Interest

The authors declare that the research was conducted in the absence of any commercial or financial relationships that could be construed as a potential conflict of interest.

## Publisher's Note

All claims expressed in this article are solely those of the authors and do not necessarily represent those of their affiliated organizations, or those of the publisher, the editors and the reviewers. Any product that may be evaluated in this article, or claim that may be made by its manufacturer, is not guaranteed or endorsed by the publisher.

## Abbreviations

UC: Ulcerative Colitis
IBD: Inflammatory bowel disease
5-ASA: 5-aminosalicylic acid
TCM: Traditional Chinese medicine
CAMs: Complementary and alternative medicines
CD: Crohn's disease
IL: Interleukin
TLR: Toll-like receptor
NF-κB: Nuclear factor κB
TNF-α: Tumor necrosis factor-α
Th: T helper type
CpG-DNA: Non-methylated bacterial DNA
MyD88: Myeloid differentiation factor 88
IRAK: IL-1 receptor-related kinase
SCFAs: Short-chain fatty acids
DSS: Dextran sulfate sodium
SYK: Spleen tyrosine kinase
CARD9: Caspase recruitment domain family member 9
TNBS, 2, 4: 6-trinitrobenzene sulfonic acid
OXA: Oxazolone
SASP: Sulfasalazine
FMT: Fecal microbiota transplantation
RCTs: Randomized controlled trials
DAI: Disease activity index
IFN-γ: Interferon-gamma
MAPK: Mitogen-activated protein kinase
Akt: Protein kinase B
MPO: Myeloperoxidase
IP10: gamma-induced protein 10
CXCR3: Chemokine (cys-x-cys motif) receptor 3
STAT3: Signal transducer and activator of transcription 3.
