# Corrigendum: The Communication Between Intestinal Microbiota and Ulcerative Colitis: An Exploration of Pathogenesis, Animal Models, and Potential Therapeutic Strategies

**DOI:** 10.3389/fmed.2022.886105

**Published:** 2022-05-10

**Authors:** Yu Hu, Zhen Ye, Mingquan Wu, Yingqi She, Linzhen Li, Yujie Xu, Kaihua Qin, Zhipeng Hu, Maoyi Yang, Fating Lu, Qiaobo Ye

**Affiliations:** ^1^School of Basic Medical Sciences, Chengdu University of Traditional Chinese Medicine, Chengdu, China; ^2^Department of Pharmacy, Sichuan Provincial Orthopedic Hospital, Chengdu, China; ^3^Hospital of Chengdu University of Traditional Chinese Medicine, Chengdu, China; ^4^Guangzhou University of Chinese Medicine, Guangzhou, China; ^5^Health Preservation and Rehabilitation College, Chengdu University of Traditional Chinese Medicine, Chengdu, China

**Keywords:** ulcerative colitis, pathogenesis, intestinal microbiota, animal model, treatment strategy, fecal microbiota transplantation, probiotic, traditional Chinese medicine

There is an error in the Funding statement. The Funding number for “the National Natural Science Foundation of China” was erroneously given as “8197151950.” The correct number is “81973742”.

The authors apologize for this error and state that this does not change the scientific conclusions of the article in any way. The original article has been updated.

## Publisher's Note

All claims expressed in this article are solely those of the authors and do not necessarily represent those of their affiliated organizations, or those of the publisher, the editors and the reviewers. Any product that may be evaluated in this article, or claim that may be made by its manufacturer, is not guaranteed or endorsed by the publisher.

